# Epidemiological Study on *Salmonella* Prevalence in Sow Herds Using Direct and Indirect Detection Methods

**DOI:** 10.3390/microorganisms10081532

**Published:** 2022-07-28

**Authors:** Isabell Hollmann, Jan Berend Lingens, Volker Wilke, Christian Homann, Klaus Teich, Juhle Buch, Bussarakam Chuppava, Christian Visscher

**Affiliations:** 1 Institute for Animal Nutrition, University of Veterinary Medicine, Foundation, 30559 Hannover, Germany; isabell.hollmann@tiho-hannover.de (I.H.); jan.berend.lingens@tiho-hannover.de (J.B.L.); christian.homann@tiho-hannover.de (C.H.); bussarakam.chuppava@tiho-hannover.de (B.C.); christian.visscher@tiho-hannover.de (C.V.); 2 AniCon Labor GmbH, 49685 Emstek, Germany; teich@anicon.eu (K.T.); buch@anicon.eu (J.B.)

**Keywords:** *Salmonella*, pigs, sows, pre-harvest, foodborne pathogen, epidemiology, real-time PCR, serology

## Abstract

In piglet production, the beginning of pork production, *Salmonella* prevalence requires greater attention as having an impact on the subsequent production steps. The aim of this study was to investigate *Salmonella* prevalence in three sow herds with attached piglet rearing units. *Salmonella* prevalence was investigated either directly by boot swabs and feces or indirectly by serum samples taken during gilt integration, the peripartal period, and piglet rearing. Boot swabs and feces were analyzed by real-time PCR and subsequent microbiology. Results indicated that high biosecurity measures in sow husbandry do not necessarily result in a low *Salmonella* prevalence. Furthermore, the sow herds’ *Salmonella* prevalence should not be used to infer the situation in the associated piglet rearing. The proportion of positive boot swabs was 10.5, 3.6, and 21.3% for sows (gilts and peripartal) with an inverse situation in piglet rearing with 50.0, 63.3, and 5.8% positive swabs for farms A, B, and C, respectively. Boot swabs are suitable as a direct sampling method to gain an overview of *Salmonella* prevalence in both sows and piglets. Indirect serum antibody testing can be useful, although it should be evaluated considering age-dependent levels of antibody titres.

## 1. Introduction

*Salmonella* is a major zoonotic pathogen of concern worldwide, contributing to the main causes of human gastroenteritis in most European countries [1,2]. In the EU as well as in Germany, salmonellosis is still the second-most reported foodborne zoonosis [1,3], although the number of cases of salmonellosis in human has decreased over the past decade [3]. In 2020, there were still 52,702 laboratory-confirmed cases of *Salmonella* infection reported in the EU [1] and 8743 confirmed cases of human salmonellosis in Germany [3]. Extensive research on the epidemiology and control of *Salmonella* in pigs in general has been conducted in the EU to date [1]. The prevalence of positive samples of *Salmonella* spp. was 28% (15,656 positive samples, *n* = 56,008) of pigs based on data reported by 10 member states in the EU in 2020 [1]. The German Quality Assurance System for the food chain, the so-called QS *Salmonella* monitoring, presents comprehensive information about farms’ situations at the primary production level by dividing fattening farms into categories I (0–20%), II (20–40%), or III (>40%) according to their proportion of serological positive samples based on a standardized sampling scheme [4,5]. In Germany, the number of farms with more than 40% of serological positive tested finishing pigs for *Salmonella* amounted to 1.6% in 2021 [6]. This represents a 70% decrease since monitoring began 20 years ago [6], yet this does not appear to be sufficient to prevent an introduction into the food chain. In accordance with the European Food Safety Agency (EFSA) and European Centre for Disease Prevention Control (ECDC) [1], the important species in the genus, *Salmonella* enterica, is one of the leading causative agents of human infections in Europe, which includes Germany. In the past few years, *S*. Typhimurium, *S*. Derby, and *S*. serovar 4,[5],12:i:- (a monophasic variant of *S*. Typhimurium) have been the most commonly detected serovars in pigs in EU countries [1], but there are still reports of the detection of *S*. Rissen, *S*. Infantis, *S*. Enteritidis, and *S*. Brandenburg [7].

Pigs can acquire *Salmonella* infection from direct contact with infected animals [8], contaminated feed, or through the environment [9]. From various research studies, it has been found that piglets moving to the fattening unit might already harbor *Salmonella* species [10]. However, most infected pigs remain clinically healthy carriers of *Salmonella* and act as asymptomatic carriers [7,11]. Those pigs infected at the end of the fattening period could cause a risk to human health [7]. Thus, control measures of *Salmonella* infection at the level of primary production among all age groups reduce the incidence of disease in fattening pigs and decrease the risk of spreading the pathogen into the food chain [4]. A pig’s *Salmonella* status can be monitored by serological testing on blood samples [12] or, more commonly, by the bacteriological analysis of collected feces as the standard method at either the farm or the slaughterhouse [13]. Mesenteric lymph nodes, obtained solely from carcasses, can also be used for studying *Salmonella* infection [14]. Swab samples are not collected quite as frequently from the pigs’ environment [13] and from carcasses [8,10].

Since estimating the prevalence and identifying infected pig herds to apply increased control measures depends on the sampling method, it is important to select a sensitive sampling method for all stages of pig production [15,16]. To test this, extensive sampling is primarily required to define parameters [17]. Currently, the duration and costs of the sampling regime must be minimized to make it viable on a large scale. For this reason, the PCR method might become of greater interest [18]. There is also a necessity to determine the sensitivity of different sampling methods to one another in order to advise risk managers and the pig industry. Nowadays, various sampling methods are used in the pig industry and by researchers to investigate *Salmonella* in swine husbandry, but these are mainly focused on the end of pre-harvest [13]. Individual sampling using *Salmonella* seroprevalence with the cut-off values of optical density (OD) has been widely used [12,19]. However, blood collection requires the animals to be restrained and affects their welfare [20]. Nonetheless, various other methods are used to sample the environment of the pig herds, e.g., boot swabs. Such alternative methods can be taken in a noninvasive way, are safe and simple, and reasonably priced [20].

Piglets of sow herds, especially of those herds purchasing replacement gilts externally, might have a decisive impact on the infection incidence for the subsequent fattening units [21,22,23,24]. There are limited data on *Salmonella* prevalence in sow herds [14,25,26]. Therefore, the present field study was conducted using different sampling methods of *Salmonella* with the aim of describing the epidemiological situation in piglet breeding farms while comparing the sampling methods in order to derive recommendations for intervention.

## 2. Materials and Methods

### 2.1. Ethical Statement

The animal experiments were carried out in accordance with the rules and regulations of the German state approved by the Ethics Committee of Animal Testing of LAVES and LALLF (Lower Saxony State Office for Consumer Protection: reference 33.8-42502-05-20A557 and State Office for Agriculture, Food Safety and Fisheries Mecklenburg–Vorpommern: reference 7221.3-2-018/20). Data were generated as part of a project funded by the Federal Ministry of Food and Agriculture (Rye-SaFe, 2813IP026).

### 2.2. The Farms and Animals

**Figure 1:**
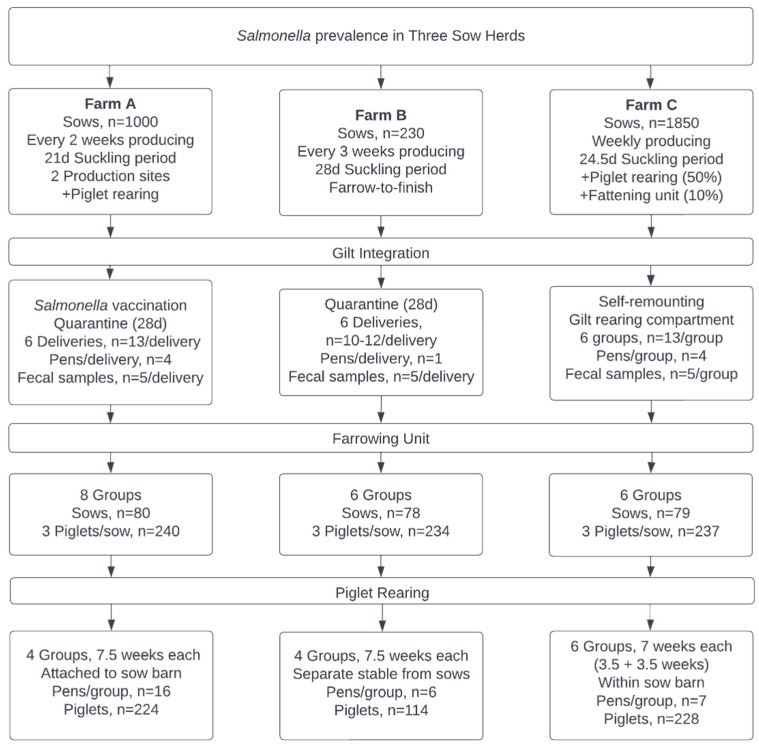
Overview of the participating sow herds according to the examined subunits.

The field study was conducted from January 2021 to May 2022 in three piglet breeding herds in northern Germany. They participated voluntarily in the field study due to recurrent *Salmonella* findings in the subsequent fattening units. Gilts on farm A were two-shot vaccinated against an attenuated *S*. Typhimurium strain (Salmoporc^®^, Ceva Tiergesundheit GmbH, Düsseldorf, Germany) during integration; vaccination was administered subcutaneously two weeks after arrival and a booster vaccination administered after another four weeks. In total, the three herds provided about 3100 breeding sows. A total of *n* = 1770 animals were sampled and a number of *n* = 1534 boot swabs, *n* = 265 fecal samples, and *n* = 2220 blood samples were tested for *Salmonella*. Figure 1 displays the three sow herds and the basis of which samples were taken in each unit.

### 2.3. Diets

Sows and piglets in herds A and B received dry purchased compound feed, which was either ground or in pelleted form. Sows and piglets in herd C additionally received purchased compound feed supplied in liquid form, except for piglets in the first three and a half weeks in the rearing unit when it was supplied dry in pelleted form. Each farm had its own feed supplier.

### 2.4. Biosecurity Check

At the beginning of the field study, a scientific risk-based and independent scoring system was carried out in order to evaluate the on-farm biosecurity. Therefore, a publicly available, standardized protocol was used (https://biocheck.ugent.be/en, accessed on 23 July 2020) which recorded hygiene status and management measures of the participating farms. The questionnaire, originally developed by Laanen et al. [27], was divided into external and internal biosecurity, which in turn were subdivided. The external biosecurity, i.e., the contact of the farm with external conditions, consisted of the following sub-units (A–F): purchase of animals and semen; transport of animals; carcasses and excrements; supply of feed, water, and objects; human traffic; vermin and bird control; location of farm. Internal biosecurity (G–L) covered on-farm disease management; farrowing and suckling period; nursery unit; fattening unit; measures between production units; cleaning and disinfection [28]. All answers given in the questionnaire were collected and expressed as a percentage, with a maximum of 100% to be achieved. Thereby, an overview of the farm’s biosecurity level in national and international comparison was provided.

### 2.5. Experimental Design

The animals, which were individually sampled, were randomly chosen for each sub-trial. In total, 226 gilts, 237 peripartal sows, 711 suckling piglets, and 596 ready-to-sell piglets were chosen. Farm differences in the number of groups included in the study and thus differences in the number of samples were due to different conditions on the farms. In each of the three production subunits (gilts, peripartal, piglets), parallel status examination of several cohorts was carried out. During the study, feed samples of the three subunits were taken in order to test for *Salmonella*.

#### 2.5.1. Gilt Integration

**Figure 2:**
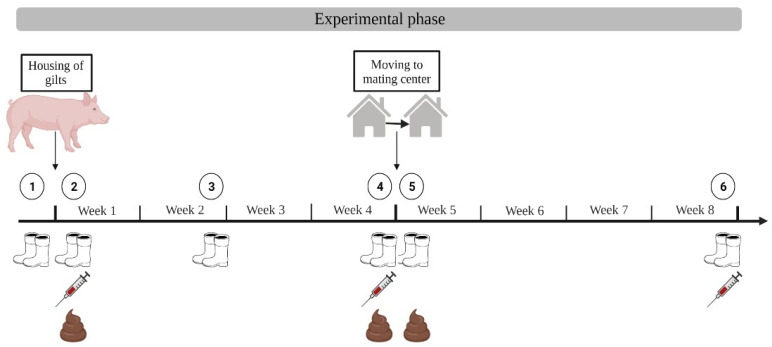
Scheme of investigation for gilt integration with sampling time points ➀–➅. Total number of samples: boot swabs *n* = 324, feces *n* = 265, blood *n* = 676 (Created with BioRender.com, accessed on 9 June 2022).

In the gilt integration, six deliveries of gilts every nine weeks were monitored on farms A and B (January 2021–March2022). On farm C, six cohorts of in-house bred gilts were monitored (February 2021–August 2021). Each cohort was sampled over a period of eight weeks. For individual testing, 78, 70, and 78 gilts were chosen on farms A, B, and C, respectively. Investigations started with boot swab samples of the cleaned and disinfected pens before the gilts were moved in. In herd C, investigations started four weeks before moving the gilts to the mating center. Investigations on all farms ended four weeks after moving the gilts to the mating center. Boot swabs were taken on a pen basis. Of those gilts which were blood sampled, five individuals were also selected for fecal sampling. The number of boot swab samples summed up to 144, 36, and 144 for farms A, B, and C, respectively. The number of fecal samples summed up to 90, 85, and 90 for farms A, B, and C, respectively. The number of blood samples summed up to 234, 208, and 234 for farms A, B, and C, respectively. The six cohorts of gilts on each farm followed the same scheme of investigation as shown in Figure 2.

#### 2.5.2. Peripartal Period

**Figure 3:**
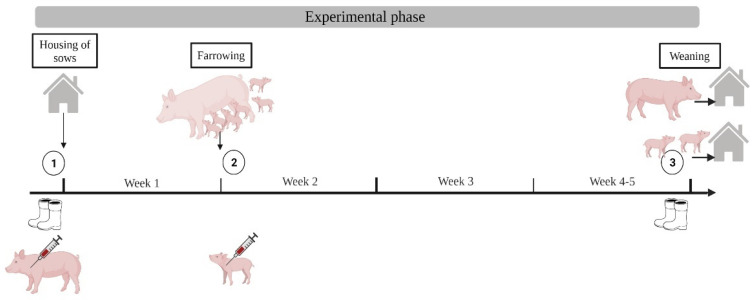
Scheme of investigation for the peripartal period with sampling time points ➀–➂. Total number of samples: boot swabs *n* = 476, blood *n* = 948 (Created with BioRender.com, accessed on 9 June 2022).

In the peripartal period, four cohorts of sows on farm A (March 2021–May 2021 and October 2021–December 2021) and six cohorts of sows each on farm B (February 2021–September 2021) and farm C (February 2021–June 2021) were examined in the farrowing unit. For individual testing, 80, 78, and 79 peripartal sows were chosen on farms A, B, and C, respectively. Sampling started before sows were moved to the cleaned and disinfected pens and ended with the weaning of the piglets. Therefore, the experimental phase differed according to the farms’ suckling period. Boot swab samples were taken of the sows’ farrowing pens. The sows, which were moved into the same pens, were blood sampled. Blood samples of new-born piglets were taken once within 24 h of farrowing. Blood was taken from three piglets in each of 80 litters in herd A, 78 litters in herd B, and 79 litters in herd C, respectively. The number of boot swab samples summed up to 160, 160, and 156 for farms A, B, and C, respectively. The number of blood samples summed up to 320, 312, and 316 for farms A, B, and C, respectively. The groups in the peripartal period followed the scheme of investigation shown in Figure 3.

#### 2.5.3. Piglet Rearing

**Figure 4:**
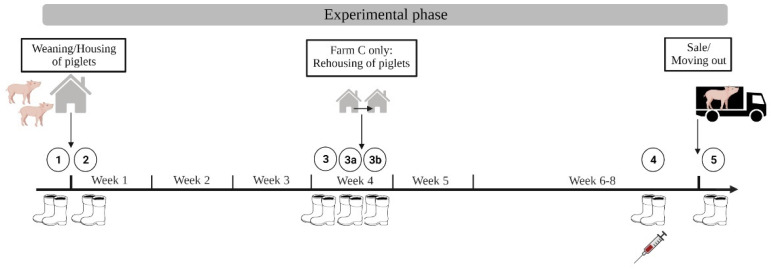
Scheme of investigation for piglet rearing with sampling time points ➀–➄ for farms A and B and two additional time points (3a and 3b) for farm C. Total number of samples: boot swabs *n* = 734, blood *n* = 596 (Created with BioRender.com, accessed on 9 June 2022).

In the piglet rearing unit, four cohorts of piglets on farm A (November 2021–March 2022), as well as on farm B (September 2021–May 2022), and six cohorts of piglets on farm C (February 2022–April 2022) were investigated. For individual testing, 224, 144, and 228 ready-to-sell piglets were randomly chosen on farms A, B, and C, respectively. Sampling of cleaned and disinfected pens started prior to moving the piglets to the rearing unit and ended after the piglets were moved out of the rearing unit. The sampling was carried out at five time points on farms A and B, whereas on farm C samples were taken at seven time points due to rehousing of the piglets after three and a half weeks of rearing. The two additional time points for boot swab samples on farm C displayed the cleaned and disinfected pens before and after rehousing the piglets to those pens. Boot swab samples were taken on a pen basis. Blood samples were only taken at the end of rearing (10–11 weeks of age) to avoid detecting antibodies at an earlier stage [29,30]. The number of boot swab samples summed up to 320, 120, and 294 for farms A, B, and C, respectively. The number of blood samples summed up to 224, 144, and 228 for farms A, B, and C, respectively. All groups followed the scheme of investigation shown in Figure 4.

### 2.6. Collection of the Samples

For detecting *Salmonella* in the pigs’ environment, boot swabs were taken on a pen basis. To avoid contamination, using a pair of disposable gloves, the sock swab (HygoStar, Franz Mensch GmbH, Buchloe, Germany) was pulled over a boot pre-covered with a plastic overshoe (WDT, Garbsen, Germany). The pen to be examined was walked through according to a standardized protocol: after walking along the outer walls, it was meandered through the pen so that a large area was covered. Care was taken to ensure that the boot swab was in contact with the ground at all times and to deliberately step into feces. Afterwards, the boot swab was removed, put into a plastic bag, and sent to the laboratory (AniCon Labor GmbH, Veterinary Diagnostic Laboratory, Höltinghausen, Germany) within 24 h for investigation.

Fecal samples were collected during spontaneous defecation, with approximately 10 g of feces transferred to a sample container using clean disposable gloves. Fecal samples were also sent to the laboratory for *Salmonella* investigation.

Blood samples were obtained from the *V. jugularis externa* of sows and from the *V. cava cranialis* of the newborn piglets [31]. The blood was collected in tubes with coagulation activator (Sarstedt Serum Monovette^®^, Sarstedt, Nümbrecht, Germany). After 6 to 12 h of sampling, blood samples were centrifuged at 3000 rpm for six minutes, serum was separated, cooled, and sent to the laboratory the same day and was analyzed the subsequent day.

### 2.7. Salmonella Detection

The study was conducted in cooperation with AniCon Labor GmbH, Veterinary Diagnostic Laboratory.

#### 2.7.1. Direct Detection Method

For detecting *Salmonella,* the environmental boot swab samples, individual fecal samples, and feed samples were analyzed directly using the PCR detection method KYLT^®^ *Salmonella* spp. (AniCon Labor GmbH, test authorization FLI-B 656, sensitivity and specificity 100%). For the molecular biological investigation, the samples were enriched in peptone water for 16 to 18 h (37 °C) and then analyzed by real-time PCR. In a second step, samples with positive PCR test results were cultured (ISO 6579-1 [32]) and typed according to the Kauffman–White scheme. Therefore, after pre-enrichment in peptone water, samples were selectively enriched on Modified Semi-Solid Rappaport-Vassiliadis (MSRV) agar for 24 to 48 h (41.5 °C) and then sub-cultured on Rambach- and Xylose-Lysin-Desoxycholat (XLD) selective nutrient medium for 24 h (37 °C). Cultures were assessed macroscopically on selective agar. In addition to the XLD selective medium, Rambach agar was chosen to differentiate red-colored *Salmonella* colonies. For serotyping, colonies were sub-cultured on blood agar, followed by a slide agglutination quick test using sera (Sifin Diagnostics GmbH, Berlin, Germany) to determine surface antigens. For differentiation between the field strains and the vaccine strain, the vaccine-specific *Salmonella* Typhimurium DIVA Real-Time PCR (Kylt^®^ ST DIVA, AniCon Labor GmbH) was performed in one single case.

#### 2.7.2. Indirect Detection Method

In addition, an indirect detection method was used for serum samples detecting for *Salmonella* lipopolysaccharide antibodies of the serovars of groups B, C, D, and E using the pigtype *Salmonella* Ab ELISA, Ver. May 2018 (Indical Bioscience GmbH, Leipzig, Germany, sensitivity and specificity 100%). An OD% of >15% was considered positive by the test manufacturer, whereas the threshold of the national *Salmonella* monitoring [4] is OD 40%. For this reason, two different OD-values were used as a threshold to classify positive blood samples: OD ≥ 15% (OD15) and OD ≥ 40% (OD40).

### 2.8. Statistical Analysis

SAS Enterprise Guide (version 7.1, Fa. SAS Institute Inc., Cary, NC, USA) was used for statistical analysis. The differences in the distribution of positive *Salmonella* samples from direct and indirect determination at farm-specific level were analyzed using the chi-square homogeneity test. The chi-square homogeneity test differentiates the distribution of sample results for each time point individually. It was calculated between the swabs and each of the other methods. Assuming that OD-values were normally distributed, data were tested for significant differences for each farm and subunits using one-way analysis of variance (ANOVA) with multiple comparisons according to the Ryan–Einot–Gabriel–Welsch test. In order to compare the sampling methods, the McNemar test was used to compare the percentage of positive samples of the different sampling methods (swab, feces, and serology); *p* < 0.05 means statistically different frequencies of positive samples according to different sample types. Moreover, the Kappa Index as a descriptive measure of agreement of individual samples between two methods (in the cross tabulation) was calculated. Kappa values were evaluated as follows: <0.01 indicates no agreement, values between 0.1 and 0.4 indicate weak agreement, values between 0.41 and 0.60 indicate clear agreement, values between 0.61 and 0.80 indicate strong agreement, and values between 0.81 and 1.00 indicate almost complete agreement between the two compared methods [33].

## 3. Results

### 3.1. Biosecurity Check

The results of the Biosecurity Check [28] for each farm compared to the national average based on 152 completed surveys are shown in the Table 1: farms A and C not only had a comparably high external biosecurity standard but also high internal standards, which resulted in both having noticeably higher biosecurity than the national mean. Farm B, on the other hand, did not meet the national average of external biosecurity. In summary, the farms were assessed with farms A and C both being on a high biosecurity level and farm B showing a considerably lower biosecurity level. Germany’s biosecurity average is below the global average, which is based on more than 10,000 completed surveys. Germany scores 3% worse in terms of external security and 14% in terms of internal security [34].

### 3.2. Salmonella Prevalence

In each of the three farms’ sub-units, *Salmonella* positive samples were obtained. In total, the boot swab samples obtained 23.2% positive results, fecal samples obtained 6.0% positive results, and the serum samples obtained 68.7% and 40.0% positive results for OD15 and OD40 thresholds, respectively.

#### 3.2.1. Farm A

The results of *Salmonella* prevalence on farm A are shown comparatively for direct and indirect detection methods in Table 2. The evaluation is based on *n* = 624 boot swab samples, *n* = 90 fecal samples, and *n* = 778 serum samples. While in the gilt integration, 21.5% of all boot swabs (*n* = 144) in herd A were positive tested for *Salmonella* but none of the individual fecal samples (*n* = 30 per time point = 90) tested positive. The proportion of positive boot swabs increased during the eight weeks of investigation and peaked after four weeks. Vaccination of gilts in herd A is displayed by the sudden significant increase in OD-values from week 0 to 4. In the peripartal period, only 1/160 swabs was positive, which was obtained at weaning. The results of the sows’ serum samples (*n* = 80) did not differ significantly (*p* > 0.05) from those of the new-born piglets (*n* = 240). In the piglet rearing, 50.0% positive boot swabs were obtained in total (*n* = 320). When looking at the different time points, one can see that the proportion of positive swabs pre-housing was similar to that at the time of moving piglets out. The peak of positive swabs was obtained at the midpoint of piglet rearing. The serum samples (*n* = 224) at the end of rearing resulted in 40.2% OD15-positive and 17.4% OD40-positive samples. In farm A, the chi-square distribution of results between boot swabs and each of the other methods differed significantly at any time point (*p* < 0.05) except for results of the gilt integration at time point two between boot swabs and OD40, at time point five between boot swabs and fecal samples, and for swabs and OD15 in the rearing unit.

#### 3.2.2. Farm B

The results of *Salmonella* prevalence on farm B are shown comparatively for direct and indirect detection methods in Table 3. The evaluation is based on *n* = 316 boot swab samples, *n* = 85 fecal samples, and *n* = 664 serum samples. In the gilt integration, only 5.6% of all boot swabs (*n* = 36) and 3.5% of fecal samples were positive. At time point five, it was not possible to obtain feces from all gilts. Therefore, the total number of fecal samples decreased at that time point. Only one positive boot swab on farm B was found after housing the gilts when also one positive fecal sample occurred. Another positive boot swab was obtained two weeks later but not in week four anymore, while another two positive fecal samples occurred. As one of the 70 gilts died a few days after the first blood sample, the total number of blood samples decreased to *n* = 208. OD-values increased in week 4 but decreased in week 8. On this farm, the distribution of results per time point did not differ significantly (*p* < 0.05) for boot swabs and OD40 at time points 2 and 6 or for boot swabs and feces. In the peripartal period, 3.1% positive swab samples (5/160) were found, of which four positive swab results were obtained in cleaned and disinfected farrowing pens. Three quarters of the sows’ blood samples (*n* = 78) were considered positive according to OD15, while only 18.6% were positive for OD40. The proportion of colostrum-derived OD15-positive serum samples of the new-born piglets (*n* = 234) was significantly higher (*p* = 0.0002) than those of the sows but the proportion was not significantly different for OD40. The chi-square distribution of swabs and serum samples differed significantly (*p* < 0.05). In the rearing unit on farm B, the greatest percentage of positive swab samples was obtained in a farm comparison. In total, out of 120 swabs, 63.3% were positive. Similar to farm A, a large proportion of the swabs was positive in the cleaned and disinfected pens at the beginning. In addition, the peak of positive swabs was seen at the midpoint of rearing. At the end of nursing, both boot swabs and serum samples (*n* = 120) were considered positive in an even higher proportion compared to farm A. Serum results on farm B (*n* = 144) were the highest in farm comparison with 77.1% OD15-positive and 54.2% OD40-positive. The chi-square distribution of swabs and OD40 in the piglet rearing differed significantly (*p* < 0.05).

#### 3.2.3. Farm C

The results of *Salmonella* prevalence on farm C are shown comparatively for direct and indirect detection methods in Table 4. The evaluation is based on *n* = 594 boot swab samples, *n* = 90 fecal samples, and *n* = 778 serum samples. In the gilt integration, 30.6% of all boot swab samples (*n* = 144) were positive and 14.4% of fecal samples were positive within the gilt integration. The peak of positive boot swabs (75.0%) and fecal samples (26.7%) in this herd was reached after moving the gilts to the mating center. The OD-value of gilts increased steadily, so that after eight weeks nearly all gilts were classified positive for OD15 and OD40. The chi-square distribution did not differ significantly between boot swabs and OD40 results at time points 2 and 4, nor between boot swabs and feces at the end of quarantine. In the peripartal period, herd C yielded the highest proportion of positive swab samples with 12.8% (20/156) in the farm comparison, of which the greater proportion (17/20) was obtained at weaning. According to the swabs, the highest proportion of positive serum results of the three herds was found in herd C: 86.1% were OD15-positive and 67.1% were OD40-positive. The average OD-values of both sows (*n* = 79) and piglets (*n* = 237) were significantly higher (*p* < 0.0001) compared to farms A and B. Similar to farm A, the sows and piglets’ results of serum samples did not differ significantly (*p* > 0.05). The chi-square distribution of swabs and serum samples differed significantly (*p* < 0.05). In the piglet rearing, the lowest *Salmonella* prevalence, with only 5.8% positive swabs of all swab samples (*n* = 294), were obtained. Different to farms A and B, no boot swab was positive in cleaned and disinfected pens. Positive swab samples peaked after housing the piglets, which was seen earlier than on farms A and B. After moving the piglets out of the stable, no positive swab was obtained. Simultaneously, the proportion of positive serum samples (*n* = 228) was also far below the other two farms: 4.4% OD15-positive and 0.9% OD40-positive.

#### 3.2.4. Feed Samples

In addition to the results described above, 59 feed samples were directly tested for *Salmonella*. All of those feed samples were PCR negative. For this reason, results are only mentioned for completeness.

### 3.3. Salmonella Serovars of PCR-Positive Samples

**Figure 5:**
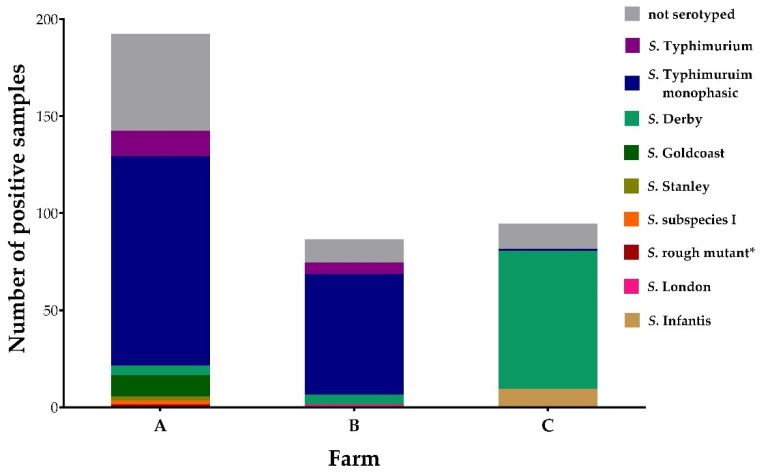
Number of PCR-positive *Salmonella* detections from boot swabs and feces, and thereof the proportion of culture positive samples classified according to serovars. * Mutant of *S*. Typhimurium with altered lipopolysaccharide O-antigen.

In total, of all 372 positive PCR results, 297 samples could be serotyped (79.8%). Divided by farms A, B, and C, the percentage of serotyped samples amounted to 74.0, 86.0, and 86.2, respectively. Positive PCR results and those that could be cultured and serotyped are shown in Figure 5. In detail, the number of each serovar found on the different farms and sub-units can be found in Supplementary Table S1. One *S*. Typhimurium positive sample isolated from the gilts on farm A was compared to the farm’s vaccine strain (Salmoporc^®^) and was confirmed as such.

### 3.4. Distribution of Frequencies and Agreement of Diagnostic Methods

The distribution of frequencies of positive test results of the different sampling methods (boot swab and feces, serum OD15 and OD40) was compared using the statistical test according to McNemar. In all cases, a significant difference was found (*p* ≤ 0.0023), which is shown in Table 5. The agreement of the different diagnostic sampling methods between the row and column of the crosstab showed the following: there was a strong agreement for gilts’ fecal samples and the sensitive threshold OD15 as well as for boot swab samples and OD15 samples in the peripartal section (Kappa ≤ 0.8). Clear agreement was seen for samples of the boot swab and OD15 method in the gilt integration (Kappa ≤ 0.6). Only weak concordance was found for the boot swab method and serum OD40 in the gilt integration and peripartal section, as well for feces and serum OD40 in the gilt integration (Kappa ≤ 0.4). There was no agreement for boot swab sampling and fecal sampling or for boot swabs and OD15 and OD40 in the piglet rearing (Kappa < 0.01). Due to the *Salmonella* vaccination of gilts on farm A, Supplementary Table S2 shows Kappa and McNemar values excluding the results of time points 4 and 6 of farm A. The Kappa values are lower but remain within the evaluation limits, giving the same agreement as shown in Table 5.

## 4. Discussion

The European Union has set itself the goal of combating *Salmonella* and other foodborne zoonotic pathogens at the level of primary production (EC No. 2160/2003). Member States’ monitoring programs are mainly conducted at the stage of fattening units but not at the stage of piglet production [19]. The present study investigated the epidemiological situation and evaluation of different sampling methods of *Salmonella* in three piglet breeding farms.

### 4.1. Epidemiological Situation on the Three Farms

In order to gain a realistic picture of *Salmonella* prevalence on the studied farms, several cohorts were examined over a period of several months. Thus, attention was paid to the seasonal influences described in the literature [35].

#### 4.1.1. Epidemiological Situation among the Gilts and Sows

In general, *Salmonella* findings were more frequent in the gilts compared to sows in the peripartal period. When comparing the serum results of the examined gilts with those of the late gestating sows, we gain a consistent picture for each farm. In herd A, the largest proportion of positive swabs in the gilt integration was typed as *S*. Typhimurium. After conformity of a single *S*. Typhimurium positive swab with the vaccine strain by using the DIVA real-time PCR examination, we assumed that at least some of the *S*. Typhimurium positive samples on this farm represented the vaccine strain. Contrary to this, there were no positive fecal samples, which could be attributed either to a non-existent infection, or more likely to the success of vaccination. Wales and Davies [36] compared several studies of *Salmonella* vaccination in their review and concluded that vaccination can reduce *Salmonella* shedding. Bearson et al. [37] found that *Salmonella* shedding was significantly reduced after vaccination. as was tissue colonization. When Buch et al. [38] investigated *Salmonella* prevalence before and after implementing vaccination, they found a significantly increased proportion of environmental samples for one of three farms after vaccination of sows. Using the Salmoporc^®^ vaccine for sows and piglets, van der Wolf et al. [39] found reduced but not absent environmental *S*. Typhimurium shedding in the four-year monitoring after the implementation of vaccination. While several positive results on farm A among gilts were attributed to the vaccination, the results of farm B, the one with the lowest biosecurity level, did not indicate a florid phase of shedding over the eight-week experimental phase. Even though the positive fecal samples on farm B in weeks 0 and 4 were considered as evidence for active shedders among the gilts, the amount of *Salmonella* did not seem to be sufficient to trigger a generalized infection [40,41,42]. This was underlined by the negative swab results in the further course of investigation. Farm C showed the highest *Salmonella* prevalence for gilts as well as for sows compared to the other two farms, despite the high biosecurity level and in-house breeding. In contrast to Farm A, where vaccination was shown to be the reason for a similar high proportion of positive serum samples, we attributed the antibody increase in gilts on farm C to a native *Salmonella* infection. Seroconversion is known to occur with a time delay [43]. The findings in farm C obtained positive boot swabs from the animals’ environment together with the positive fecal samples in weeks 4 and 5 led to this assumption.

Only farm C obtained a noteworthy number of positive swabs at weaning, again supporting the hypothesis of highest prevalence in this farm’s sow area. Nollet et al. [25] investigated *Salmonella* prevalence on three Belgian farrow-to-finish farms and found *Salmonella* at a low prevalence of <10% in late gestation, farrowing, and lactation. Similar findings were made by Buch et al. [38] in the sow area. Funk et al. [44] also found that *Salmonella* was lower among farrowing sows (0 to 9.1%) than among gestating sows (17.4 to 41.3%) in the same herd.

#### 4.1.2. Epidemiological Situation among the Piglets

It is known that sows’ *Salmonella* antibodies are transferred to their suckling piglets via colostrum [45]. By comparing the sows’ blood samples with those of the 24-hold piglets, an adequate colostrum supply in all herds could be concluded. As Roesler et al. [46] suggested, a transmission of maternal *Salmonella* antibodies may lead to an effective reduction in *Salmonella* prevalence in the subsequent piglet rearing. It can be assumed that the relatively high OD-values of both peripartal sows and suckling piglets on farm A are due to vaccination. Although a low *Salmonella* prevalence in the sows’ unit on farms A and B combined with colostral immunity was concluded, the high number of positive swabs in the rearing unit (50.0% and 63.3%, respectively) indicated a high *Salmonella* prevalence. Our results were even higher than those that Buch et al. [38] observed in the rearing area of SC farms with up to 40.8% positive environmental samples. When they compared the proportion of positive swabs from the rearing unit to the sow and gilt area, it was also significantly higher for the rearing unit. This phenomenon is also described by Kranker et al. [47] as a peak of *Salmonella* shedding in the piglet nursery. We found the highest proportion of positive swabs on farms A and B to be at the midpoint of piglet rearing. This coincides with the drop-off of maternal antibodies, which is known to decrease between the fourth and eighth week, making piglets vulnerable for a *Salmonella* infection [45,48]. The active immunization was displayed by the serum samples at the end of the rearing period with average OD-values of 20.0 and significantly higher average OD-values of 51.8 in farms A and B, respectively. Schulte zu Sundern et al. [49] sampled piglets of the same age at the end of rearing and found lower values: In two of four farms, average OD-values were just below our findings for farm A (19.3). On the remaining two farms, they found OD-values up to 3.8, which were similar to those we found on farm C.

Similar to what Schulte zu Sundern et al. [50] observed, an inverse phenomenon was found on farm C: even though the comparatively highest *Salmonella* prevalence among the gilts and sows was found, surprisingly, it was not the same for the nursery unit. It seems the significantly higher antibody titres alone may have not led to the low rate of positive samples on farm C. It can be assumed that the combination with successful separation of the functional areas led to the low rate of positive samples. Hill et al. [51] referred to it as a “delicate balance” between immunity and infection when looking at the mixed evidence found in this context. Even after rehousing the piglets, which poses another risk due to repeated stress [52], only a few positive swab samples were found during the investigation period. In contrast to farm C, farms A and B obtained positive swabs in the cleaned and disinfected pens. They were found in a quantitatively similar dimension as at the time of moving piglets out of the nursery unit. At this stage, one can still see the chance of improvement. Similar to fattening pigs and poultry, the operational procedures in the nursery unit offer opportunities to enhance hygiene using the “all-in/all-out” procedure [53], which in most cases is not possible for breeding sows. With strict adherence, infection chains could be successfully interrupted and therefore *Salmonella* pressure reduced [54,55].

The results of the study indicate that the biosecurity level, based on the UGent questionnaire [28], is not adequate alone to infer *Salmonella* prevalence on sow farms. It does not reflect the widespread assumption that high biosecurity and hygiene levels, which are included in the scoring system, are sufficient to lower *Salmonella* prevalence [56]. Our findings, particularly in the case of sow husbandry, are supported by other studies [57,58] which found that improved hygiene measures alone are not sufficient to significantly reduce *Salmonella*. Moreover, the present study indicated that piglet rearing, which is often associated with sow husbandry, must be considered separately in terms of *Salmonella* prevalence. Two previous retrospective studies [38,50] both used the same farm classifications based on the seroprevalence of ready-to-sell piglets over a longer period of time. Hence, farms were classified as “*Salmonella*-conspicuous” (SC) and “*Salmonella*-inconspicuous” (SI). One of the studies confirmed the classification by repeated serological testing of rearing piglets [38]. However, the other study tested sows from these farms serologically and tests revealed the opposite: higher OD-values in the sows from the SI farms [50]. This raised the question as to whether the classification made on the basis of the ready-to-sell piglets represents a realistic picture of the sow herd. Based on our own results, as well as the results of the previously mentioned studies [38,50], it can be assumed that the *Salmonella* status of the ready-to-sell piglets appears to be an indicator for the implementation of reduction measures, which seem to be even more effective at this production stage.

### 4.2. Salmonella Serovars

Information about the serovar might not only help in terms of epidemiological research but also in risk assessment. Several studies indicate that shedding is serovar-dependent: *S*. Typhimurium and *S*. Derby as two of the most common serovars among pigs [7] are far more likely to enter the state of intermittent non-shedding [42]. Moreover, the study by Pires et al. [59] suggests that the duration of shedding is serovar dependent. They concluded a shorter time of survival for *S*. Derby.

Besides a higher proportion of positive swabs in the piglet rearing of farms A and B, more and different *Salmonella* serovars compared to the sow barn on all three farms were found, which confirmed the findings of Kranker et al. [47]. In general, rearing units with a lot of human and animal traffic display a vulnerable point in the production process [60]. This again emphasizes not only the importance of cleaning and disinfection, but also the need to separate the area of sow husbandry from the rearing unit in order to avoid transmission between production areas. Similar to our findings, Funk et al. [44] were not able to demonstrate a serovar-specific correlation between culture-positive sows and their piglets. In contrast, Casanova-Higes et al. [61] found that 89% of the serovars found in sows were also found in weaned piglets in the same production site.

The rearing area of farm A was located in the sow house building with strict biosecurity measures when entering. The results of the cultural examination of the swabs supported the successful implementation of the hygiene measures. On farm B, the occurrence of serovars other than in the sow area was the least surprising, as the rearing pens were located in a separate place with a decentral entrance, located in old buildings. The introduction of pathogens via humans or others such as rodents is not considered as an improbable source [62]. Surprisingly, even though the piglets on farm C were first housed adjacent to the farrowing sows, there seemed to be low transmission of *Salmonella* serovars, supporting good hygiene practices. Positive samples from the sow area were classified as *S*. Derby, whereas only two samples in the adjacent rearing unit were positive for *S*. Derby and the majority serotyped as *S*. Infantis. 

### 4.3. Comparison of the Salmonella Detection Methods

The results of the present study indicate that the direct detection method of real-time PCR analysis of boot swab samples is particularly suitable for sow husbandry and piglet rearing to provide rapid evidence of an acute *Salmonella* infection. In addition to the direct PCR examination of the sample, the subsequent cultural examination was successful in at least 74% of the cases. Several studies describe the PCR assay as more sensitive than the more frequently used microbiological method: Malorny et al. [63] state that the PCR assay is highly accurate compared to the cultural method, with no false positive or false negative results. Koyuncu et al. [64] suggest that false positive PCR results are more likely to be seen as false negative bacteriological results. We decided to carry out the cultural examination of a positive PCR result to meet the limitation of the PCR assay, as it is not able to differentiate between the viable and dead pathogen [18] and offered the chance to follow epidemiological processes in more detail. The advantage of the boot swab sampling method is not only the timely exact detection of the *Salmonella*, but also the non-invasive method compared to the blood sample. Moreover, with only one boot swab, a large area of the animals’ environment can be covered. Visscher et al. [10] successfully used the boot swab method with subsequent microbiological examination to detect *Salmonella* in the environment of four fattening pig units. In addition, van der Wolf et al. [39] used boot swabs in their long-term monitoring and found them to be more sensitive than pooled fecal samples. 

Serological testing alone may lead to false epidemiological interpretation. Moreover, in order to classify serum samples, it should be considered that there is a correlation between the age of the tested animals and the level of antibody titre. The study by Wilhelm et al. [15] indicated that the older the animals become, the more likely it is to find higher OD-values. In our study, the OD15 threshold seemed to be useful for evaluating the epidemiological situation of the ready-to-sell piglets. The threshold was given by the ELISA test kit manufacturer, which might be different when using an alternative test kit [10,50]. The study by Seybold et al. [16] matches our findings and suggests using a more sensitive threshold than OD40 for ready-to-sell piglets or, even better, using the bacteriological examination. In the present study, the chi-square distribution of positive sample results showed greater agreement between the OD40 classification and boot swabs for gilts than for sows one week before farrowing, although the OD-values were beneficial for validating the boot swabs. A combination of indirect and direct detection methods seems to be useful in subclinical infected herds.

The sampling times of individual fecal samples were explicitly chosen to be in a phase with increased stress due to transport and rehousing to increase the probability of obtaining positive fecal samples [52,65]. This was despite only a low proportion of positive fecal samples occurring in two of three herds. Fecal results of herd A were previously discussed and believed to be caused by vaccination. *Salmonella* is excreted intermittently [66,67]; therefore, individual fecal samples alone would have led to an underestimation of actual prevalence, as shown in the findings of Funk et al. [68]. Arnold et al. [69] found an increasing sensitivity for pooled fecal samples, with 20 samples within the pool being the most sensitive. Sanchez et al. [13], who reviewed 238 studies from 23 countries using different detection methods, concluded farm-level apparent *Salmonella* prevalence was higher when using pooled fecal samples instead of individual samples. It could be argued that the boot swabs examined in the present study can not only be seen as an environmental sample but also be considered as an overview of the total excretion of a group or of one individual over a longer period of time.

## 5. Conclusions

The results of the present study indicated that high biosecurity measures alone do not guarantee a low *Salmonella* prevalence in sow husbandry. In addition, it was shown that *Salmonella* prevalence in the sow herd cannot simultaneously be equated with prevalence in the associated rearing unit, which poses a high-risk element in the complex system. Further research is needed to identify the reasons for this inverse epidemiological phenomenon. In order to gain an overview of *Salmonella* prevalence in sow husbandry and piglet rearing, environmental boot swab samples analyzed with the direct real-time PCR method can be revealing. Nevertheless, results of PCR analysis still leave room for interpretation. When using the indirect serological detection method, it should be evaluated with regard to the age of the tested individual.

## Figures and Tables

**Table 1:** Biosecurity screening, data in % (100 = highest biosecurity level).

Parameter	Farm A	Farm B	Farm C	National Average (GER)
External				
A—purchase of animals and semen	96	84	100	89
B—transport of animals, carcasses, and excrements	100	67	90	77
C—supply of feed, water, and objects	57	47	67	47
D—human traffic	100	88	100	72
E—vermin and bird control	90	60	100	72
F—location of the farm	80	0	90	52
External sum * (A–F)	90	64	92	72
Internal				
G—on-farm disease management	80	80	100	64
H—farrowing and suckling period	64	36	64	50
I—nursery unit	100	86	36	71
J—fattening unit	-	64	79	61
K—measures between production units	68	36	64	41
L—cleaning and disinfection	75	50	85	55
Internal sum * (G–L)	76	54	70	53
External + internal sum (A–L)	83	59	81	63

* External and internal sum calculated as a weighted average of the corresponding categories. “-“ not applicable.

**Table 2:** Comparing *Salmonella* positive results of farm A for direct (boot swabs and feces) and indirect (serum) detection methods divided according to gilt integration, peripartal, and piglet rearing.

Time Point			Direct		Indirect (Serum)			*p*-Value		
			Pos. Samples/%		Average OD	Pos. Samples/%				
No.	Week	Event	Bs	f		OD15	OD40	bs-f	bs-OD15	bs-OD40
			Gilt integration							
		*n*	144	90	234					
1	−1	Pre-housing	1/4.2	-	-	-	-	-	-	-
2	0	Housing	5/20.8	0/0.0	19.7	51/65.4	9/11.5	**0.0134**	**0.0001**	0.3087
3	2	Half of quar.	8/33.3	-	-	-	-	-	-	-
4	4	End of quar.	11/45.8	0/0.0	117.2	78/100.0	72/92.3	**<0.0001**	**<0.0001**	**<0.0001**
5	5	Moving to m.c.	2/8.3	0/0.0	-	-	-	0.1929	-	-
6	8	End	4/16.7	-	131.5	78/100.0	77/98.3	-	**<0.0001**	**<0.0001**
		Total gilts	31/21.5	0/0.0	-	207/88.5	158/67.5	-	-	-
			Peripartal							
		*n*	160	-	320					
1	−1	Pre-housing	0/0.0	-	58.4	67/83.8	40/50.0	-	**<0.0001**	**<0.0001**
2	1	Farrowing	-	-	47.8	188/78.3	120/50.0	-	-	-
3	3	Weaning	1/1.3	-	-	-	-	-	-	-
		Total peripartal	1/0.6	-	-	255/79.7	160/50.0	-	-	-
			Piglet rearing							
		*n*	320		224					
1	−1	Pre-housing	31/48.4	-	-	-	-	-	-	-
2	0	Housing	18/28.1	-	-	-	-	-	-	-
3	3	Midpoint	44/68.8	-	-	-	-	-	-	-
4	7	Before moving out	33/51.6	-	20.0	90/40.2	39/17.4	-	0.1044	**<0.0001**
5	8	After moving out	34/53.1	-	-	-	-	-	-	-
		Total piglets	160/50.0	-	-	90/40.2	39/17.4	-	-	-
*n*			624	90		778	778			
Total farm A			192/30.8	0/0.0		552/71.0	357/45.9			

Event: quar. = quarantine, m.c. = mating center; Direct: bs = boot swab, f = fecal; Indirect: OD = optical density, OD15/40 = OD threshold 15% or 40%. *p*-value of chi-square homogeneity test < 0.05 was considered significant (bold); “-“ not applicable.

**Table 3:** Comparing *Salmonella* positive results of farm B for direct (boot swabs and feces) and indirect (serum) detection methods divided according to gilt integration, peripartal, and piglet rearing.

Time Point			Direct		Indirect (Serum)			*p*-Value		
			Pos. Samples/%		Average OD	Pos. Samples/%				
No.	Week	Event	bs	f		OD15	OD40	bs-f	bs-OD15	bs-OD40
			Gilt integration							
		*n*	36	85	208					
1	−1	Pre-housing	0/0.0	-	-	-	-	-	-	-
2	0	Housing	1/16.7	1/3.3	35.1	53/75.7	23/32.9	0.3095	**0.0068**	0.6582
3	2	Half of quar.	1/16.7	-	-	-	-	-	-	-
4	4	End of quar.	0/0.0	2/6.7	47.8	63/91.3	35/50.7	1.0000	**<0.0001**	**0.0271**
5	5	Moving to m.c.	0/0.0	0/0.0	-	-	-	-	-	-
6	8	End	0/0.0	-	40.4	58/84.1	30/43.5	-	**<0.0001**	0.0752
		Total gilts	2/5.6	3/3.5	-	174/83.7	88/42.3	-	-	-
			Peripartal							
		*n*	160	-	312					
1	−1	Pre-housing	4/5.0	-	40.8	46/59.0	13/16.7	-	**<0.0001**	**0.0180**
2	1	Farrowing	-	-	24.6	188/80.8	45/19.2	-	-	-
3	4	Weaning	1/1.3	-	-		-	-	-	-
		Total peripartal	5/3.1	-	-	234/75.0	58/18.6	-	-	-
			Piglet rearing							
		*n*	120	-	144					
1	−1	Pre-housing	10/41.7	-	-	-	-	-	-	-
2	0	Housing	8/33.3	-	-	-	-	-	-	-
3	4	Midpoint	21/87.5	-	-	-	-	-	-	-
4	7	Before moving out	21/87.5	-	51.8	111/77.1	78/54.2	-	0.2496	**0.0021**
5	8	After moving out	16/66.7	-	-	-	-	-	-	-
		Total piglets	76/63.3	-	-	111/77.1	78/54.2	-	-	-
*n*			316	85		664	664			
Total farm B			83/26.3	3/3.5		519/78.2	224/33.7			

Event: quar. = quarantine, m.c. = mating center; Direct: bs = boot swab, f = fecal; Indirect: OD = optical density, OD15/40 = OD threshold 15% or 40%. *p*-value of chi-square homogeneity test < 0.05 was considered significant (bold); “-” not applicable.

**Table 4:** Comparing *Salmonella* positive results of farm C for direct (boot swabs and feces) and indirect (serum) detection methods divided according to gilt integration, peripartal, and piglet rearing.

Time Point			Direct		Indirect (Serum)			*p*-Value		
			Pos. Samples/%		Average OD	Pos. Samples/%				
No.	Week	Event	bs	f		OD15	OD40	bs-f	bs-OD15	bs-OD40
			Gilt integration							
		*n*	144	90	234					
1	−1	Pre-housing	0/0.0	-	-	-	-	-	-	-
2	0	Housing	4/16.7	0/0.0	23.9	48/61.5	10/12.8	**0.0336**	**0.0001**	0.7352
3	2	Half of quar.	4/16.7	-	-	-	-	-	-	-
4	4	End of quar.	9/37.5	5/16.7	38.2	48/61.5	20/25.6	0.0826	**0.0381**	0.2601
5	5	Moving to m.c.	18/75.0	8/26.7	-	-	-	**0.0004**	-	-
6	8	End	9/37.5	-	89.1	77/98.7	62/79.5	-	**<0.0001**	**<0.0001**
		Total gilts	44/30.6	13/14.4	-	173/73.9	92/39.3	-	-	-
			Peripartal							
		*n*	156	-	316					
1	−1	Pre-housing	3/3.8	-	76.9	73/92.4	55/69.6	-	**<0.0001**	**<0.0001**
2	1	Farrowing	-	-	67.4	199/84.0	157/66.2	-	-	-
3	4	Weaning	17/21.8	-	-		-	-	-	-
		Total peripartal	20/12.8	-	-	272/86.1	212/67.1	-	-	-
			Piglet rearing							
		*n*	294	-	228					
1	−1	Pre-housing 1	0/0.0	-	-	-	-	-	-	-
2	0	Housing 1	11/26.2	-	-	-	-	-	-	-
3	3	Midpoint	2/4.8	-	-	-	-	-	-	-
3a	3	Pre-housing 2	0/0.0	-	-	-	-		-	-
3b	4	Housing 2	3/7.1	-	-	-	-	-	-	-
4	6	Before moving out	1/2.4	-	3.6	10/4.4	2/0.9	-	0.7028	0.3991
5	7	After moving out	0/0.0	-	-	-	-	-	-	-
		Total piglets	17/5.8	-	-	10/4.4	2/0.9	-	-	-
*n*			594	90		778	778			
Total farm C			81/13.6	13/14.4		455/58.5	306/39.3			

Event: quar. = quarantine, m.c. = mating center; Direct: bs = boot swab, f = fecal; Indirect: OD = optical density, OD15/40 = OD threshold 15% or 40%. *p*-value of chi-square homogeneity test < 0.05 was considered significant (bold); “-” not applicable.

**Table 5:** Distribution of frequencies using McNemar test (grey) and Kappa statistic values (black) showing agreement between the boot swab, fecal, and blood sampling methods for *Salmonella* detection for gilt integration, peripartal, and piglet rearing.

Subunit			Kappa			
			Boot Swab	Feces	OD15	OD40
Gilts Integration	*p* -value McNemar test	Boot swab		−0.20	0.56	0.21
		Feces	<0.0001		0.68	0.32
		OD15	0.0023	<0.0001		-
		OD40	<0.0001	<0.0001	-	
Peripartal	*p* -value McNemar test	Boot swab		-	0.69	0.32
		Feces	-		-	-
		OD15	<0.0001	-		-
		OD40	<0.0001	-	-	
Piglet Rearing	*p* -value McNemar test	Boot swab		-	0.01	−0.15
		Feces	-		-	-
		OD15	<0.0001	-		-
		OD40	<0.0001	-	-	

“-” not applicable.

## Data Availability

The data presented in this study are available on request from the corresponding author.

## Supplementary Materials

The following supporting information can be downloaded at: www.mdpi.com/article/10.3390/microorganisms10081532/s1, Table S1. Number of serovars found in the sub-units on farms A, B, and C. Table S2. Distribution of frequencies using McNemar test and Kappa statistic values showing agreement between the boot swabs, feces, and blood sampling methods for *Salmonella* detection for gilt integration, excluding results of time points 4 and 6 of farm A due to vaccination.
