# Supplementary material for: Epidemiological Study on Salmonella Prevalence in Sow Herds Using Direct and Indirect Detection Methods
Source: Microorganisms. 2022 Jul 28;10(8):1532. doi: 10.3390/microorganisms10081532 (PMC9413226; doi:10.3390/microorganisms10081532)
Supplement: Supplementary file 1 [file microorganisms-10-01532-s001.zip › microorganisms-1796001-supplementary.pdf]

Table S1. Number of serovars found in the sub-units on the farms A, B, and C.

**Serovar**	**Farm**		
	**A**	**B**	**C**
	**(Gilts Integration/Peripartal/Piglet Rearing)**		
S. Typhimurium	12/0/1	0/0/6	-
S. Typhimurium monophasic	1/0/107	0/0/62	0/0/1
S. Derby	5/0/0	2/3/0	49/20/2
S. Goldcoast	0/0/11	-	-
S. Stanley	1/0/1	-	-
S. subspecies I	0/0/2	-	-
S. rough mutant*	0/0/1	-	-
S. London	-	1/0/0	-
S. Infantis	-	-	0/0/9
Total number per farm	19/0/123	3/3/68	49/20/12

"-" not applicable; * Mutant of *S*. Typhimurium with altered lipopolysaccharide O-antigen.

Table S2. Distribution of frequencies using McNemar test (grey) and Kappa statistic values (black) showing agreement between the boot swabs, feces, and blood sampling methods for *Salmonella* detection for gilt integration excluding results of time points 4 and 6 of farm A due to vaccination.

		Kappa			
		Boot swab	Feces	OD15	OD40
	Boot swab		-0.19	0.51	0.11
p-value	Feces	<0.0001		0.64	0.23
McNemar test	OD15	0.0017	<0.0001		-
	OD40	<0.0001	<0.0001	-	

"-" not applicable
